# DNA methylation at birth within the promoter of ANRIL predicts markers of cardiovascular risk at 9 years

**DOI:** 10.1186/s13148-016-0259-5

**Published:** 2016-09-02

**Authors:** Robert Murray, Jennifer Bryant, Phil Titcombe, Sheila J. Barton, Hazel Inskip, Nicholas C. Harvey, Cyrus Cooper, Karen Lillycrop, Mark Hanson, Keith M. Godfrey

**Affiliations:** 1 Human Development and Health Academic Unit, Faculty of Medicine, University of Southampton, Southampton, UK; 2 NIHR Southampton Biomedical Research Centre, University Hospital Southampton NHS Foundation Trust (UHSFT) and University of Southampton, Southampton, UK; 3 MRC Lifecourse Epidemiology Unit, University of Southampton, Southampton, UK; 4 Radiology, UHSFT, Southampton, UK; 5 Centre for Biological Sciences, Faculty of Natural and Environmental Sciences, University of Southampton, Southampton, SO17 1BJ UK

**Keywords:** Pregnancy, Non-coding RNA, Pulse wave velocity

## Abstract

**Aims:**

Antisense non-coding RNA in the INK4 locus (ANRIL) fixed genetic variants have consistently been linked with coronary heart disease (CHD) risk. We investigated relationships between perinatal ANRIL promoter DNA methylation and CHD risk markers in children aged 9 years. Genetic variants in the non-coding RNA ANRIL identify it as an important CHD risk locus. Increasing evidence suggests that the early life environment may act through epigenetic processes to influence later CHD risk markers such as increased arterial pulse wave velocity (PWV, a measure of arterial stiffness) blood pressure or heart rate.

**Methods and results:**

Using pyrosequencing, ANRIL DNA methylation at nine CpG sites was measured in the umbilical cord from 144 children in a UK mother-offspring cohort and related to the descending aorta PWV measured by velocity-encoded phase contrast MRI at age 9 years. Perinatal methylation was not associated with child’s later blood pressure, but higher methylation at CpG5 was associated with increased childhood PWV (*β* = 0.066 m/s/10 % methylation increase [95 % CI, 0.004 to 0.128], *p* = 0.037); 10 % decreases in methylation at CpG1 and CpG2 were associated with increased heart rate (CpG1 *β* = 1.93 [0.07 to 3.8] beats/min, *p* = 0.041; CpG2 *β* = 2.30 [0.18 to 4.41] beats/min, *p* = 0.033, accounting for potential confounding variables). The associations with perinatal ANRIL promoter methylation were independent of neighbouring fixed genetic variants.

**Conclusions:**

Our findings suggest developmental epigenetic regulation of ANRIL promoter methylation as a factor in later CHD risk in children.

**Electronic supplementary material:**

The online version of this article (doi:10.1186/s13148-016-0259-5) contains supplementary material, which is available to authorized users.

## Introduction

The 9p21 region is the strongest candidate for coronary heart disease (CHD) risk at the population level identified so far from genetic studies [1, 2]. This region contains several protein-coding genes that regulate cell cycle progression (p14ARF, p15INK4b, p16INK4a) as well as the long non-coding RNA antisense non-coding RNA in the INK4 locus (ANRIL). Recent studies have shown that genetic variants associated with CHD map onto the ANRIL gene rather than the protein coding genes and single-nucleotide polymorphisms (SNPs) in ANRIL, linked to increased risk of CHD, are associated with decreased expression of ANRIL transcripts. [3–5] The functional role of ANRIL in cardiovascular disease has also been investigated in vascular smooth muscle, where ANRIL knockdown altered the expression of genes involved in the remodelling of the extracellular matrix, suggesting that ANRIL impacts on CHD risk via the modulation of these processes [6], leading to altered vascular structure and function.

Experimental and epidemiological studies provide strong evidence that the early life environment influences later cardiovascular disease risk [7], and this has been suggested to involve the altered epigenetic regulation of gene function. Epigenetic processes, which include DNA methylation, can induce stable changes in gene expression without a change in gene sequence [8]. However, to date, there have been no longitudinal studies showing that prenatal epigenetic processes are associated with CHD risk. Thus, the aim of this study was to examine the relationship between DNA methylation levels at birth in the promoter region of ANRIL with blood pressure (BP), heart rate (HR) and pulse wave velocity (PWV) in children aged 9 years. Increased resting HR is a risk factor for CHD in males and is associated with cardiovascular death in both sexes [9–12], while increased PWV, an indicator of arterial stiffness, has been shown to be a strong predictor of atherosclerosis, cardiovascular mortality, myocardial infarction, angina, heart failure, and stroke in adulthood [13, 14]. Here, we found that ANRIL promoter methylation at birth was associated with both heart rate and arterial stiffness at 9 years of age, providing further evidence for the importance of the early life environment in influencing health in later life and suggesting that differential methylation of CpG loci within the promoter of ANRIL may provide a marker to identify individuals in early life at increased risk of CHD disease in later life.

## Methods

### Cohort

In a UK mother-offspring study (Southampton Women’s Survey, SWS [15]), maternal characteristics were ascertained before and during pregnancy and offspring measurements obtained after birth.

### Child adiposity measurement

At age 8 years, child adiposity measurements were made by dual-energy X-ray absorptiometry (Hologic Discovery, paediatric scan mode, Hologic Inc., Bedford, MA) [16]. The instrument was calibrated daily; coefficients of variation were 1.4 to 1.9 %. Follow-up of the children and sample collection/analysis was carried out under Institutional Review Board approval (Southampton and SW Hampshire Research Ethics Committee) with written informed consent. Clinical investigations were conducted according to the principles expressed in the Declaration of Helsinki.

### Pulse wave velocity measurements

At age 9 years, a subset of SWS participants was invited to attend for magnetic resonance imaging (MRI) assessment of cardiovascular structure and function. PWV was measured in the descending aorta. A phase-contrast flow-mapping sequence was acquired at the level of the pulmonary trunk in the proximal descending aorta and in the distal descending aorta above the bifurcation. A velocity-encoding gradient was applied in the through-plane direction. Right brachial blood pressure was measured immediately following the acquisition. Velocity flow curves were generated using open source software (Osirix). Descending aortic PWV was calculated using Matlab software (Mathworks, Natick, MA) and the transit time method [17] from distance between the flow acquisitions/transit time of the systolic wave front between the two flow acquisition sites.

### DNA methylation analysis

Genomic DNA was extracted from umbilical cord tissue which had been collected at birth and stored at −80 °C. DNA was bisulphite converted using the EZ DNA methylation kit (ZymoResearch, USA) (Primers: Additional file 1: Table S2). Modified DNA was amplified using Hotstart Plus DNA polymerase (QIAGEN). PCR products were immobilised on streptavidin–sepharose beads (GE Healthcare), washed, denatured and released into annealing buffer containing sequencing primer. Pyrosequencing was carried out on a Pyromark MD (Qiagen). %methylation was calculated using the Pyro Q CpG software (QIAGEN). Additional file 2: Table S1 shows CpG genomic co-ordinates.

### Statistics

Statistical analysis was performed using Stata (Statacorp, USA) versions 13.1 and 14.0. Descending aorta pulse wave velocity, heart rate, and systolic and diastolic blood pressures at 9 years were used as outcome measures. %methylation of ANRIL CpGs 1-9 were used individually as predictors, together with sex and age at MRI scan. All outcomes measures were approximately normally distributed. Linear regression models were built for each outcome with ANRIL methylation (taking one CpG at a time) as a predictor, adjusting for sex and age at MRI scan. Where regression residuals showed heteroscedasticity (Cook-Weisberg test [18]), robust estimators of standard errors were calculated and used for calculating the test statistic. If CpGs were significant predictors of outcomes (*p* < 0.05), further adjustments were made for child’s fat mass (or fat %) at age 9 years and maternal smoking during pregnancy. Results are presented as regression coefficients multiplied by 10 (*β*), representing the change in cardiovascular outcome per 10 % change in methylation, with associated *p* values and 95 % confidence intervals. DNA Methylation levels of some CpGs were highly correlated (Additional file 3 : Table S3, CpGs 1-3 and 5-7), and it was therefore inappropriate to carry out statistical corrections that assume independence, such as Bonferroni or Benjamini-Hochberg correction.

## Results

### Cohort characteristics

We studied 144 children (72 boys) (Table 1); mean heart rate was 80.2 bpm; mean descending aorta PWV was 3.4 m/s (within previously reported childhood ranges [19]); mean % and total fat mass measurements were 24.6 % and 4.8 kg, respectively; 14.7 % of mothers smoked during pregnancy. Umbilical cord ANRIL methylation levels varied greatly; for example, the 5th–95th percentile ranges for CpGs 2, 3 and 5 were 20–30 % (Additional file 2: Table S1).

**Table 1:** Characteristics of the study population

	Number	Percent or median (25th, 75th percentile)
Mother		
Pregnancy smoking status	143	
Smoker	21	14.7 %
Non-smoker	122	85.3 %
Infant		
Birth weight, kg	142	3.4 (3.1–3.8)
Sex	144	
Male	72	50 %
Female	72	50 %
%fat age 8 years	132	24.6 (20.5–29.6)
Total fat mass age 8 years, kg	132	6.6 (5.0–09.0)
Child follow-up		
Age, year	144	9.4 (9.3–9.6)
Heart rate, bpm	137	80.2 (74.0–85.4)
Systolic BP, mm Hg	135	98.0 (92.0–105.0)
Diastolic BP, mm Hg	135	58.0 (55.0–62.0)
Descending aorta PWV, m/s	137	3.4 (3.2–3.7)

### Heart rate is associated with ANRIL promoter DNA methylation

**Fig. 1:**
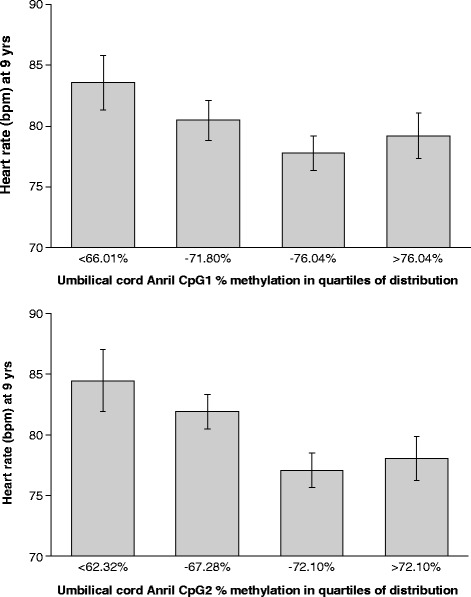
Heart rate (bpm) vs ANRIL DNA methylation at CpG1 and CpG2. DNA methylation levels were determined via pyrosequencing. The *x*-axis shows quartiles of methylation; values are means + SEM. *N* = 132

To examine whether DNA methylation levels within the promoter region of ANRIL were associated with heart rate, we analysed the methylation status of nine CpG sites within the promoter of ANRIL at birth in relation to measures of cardiovascular risk at age 9 years. Methylation at CpG1 and CpG2 correlated inversely with heart rate (*p* = 0.031, *p* = 0.02, respectively) (Table 2, Fig. 1). These associations remained significant in multivariate models that controlled for child’s total fat mass at 8 years, sex, age and maternal smoking; CpG1 *p* = 0.041, CpG2 *p* = 0.033. The total variances in heart rate explained were 7.6 and 8.6 % for CpG1 and CpG2, respectively.

**Table 2:** Heart rate (bpm) at age 9 years in relation to ANRIL promoter DNA methylation

CpG	Hg19 coordinates	*β* value	*p* value	Adjusted *p*
1	chr9: 21993721	−1.98	0.031	0.041
2	chr9: 21993697	−2.45	0.020	0.033
3	chr9: 21993694	−2.56	0.080	0.103
4	chr9: 21993654	−1.5	0.195	0.168
5	chr9: 21993645	−0.669	0.484	0.488
6	chr9: 21993638	−1.42	0.128	0.196
7	chr9: 21993629	0.096	0.938	0.936
8	chr9: 21993603	−1.18	0.288	0.402
9	chr9: 21993583	−0.626	0.642	0.463

*β* values represent a 10 % change in methylation. DNA methylation levels were determined via pyrosequencing. Linear regressions were used to compare methylation and heart rate. Adjusted *p* values are adjusted for age, sex, total fat mass at 8 years and mother smoking during pregnancy

### PWV associates with DNA methylation at the ANRIL promoter

**Fig. 2:**
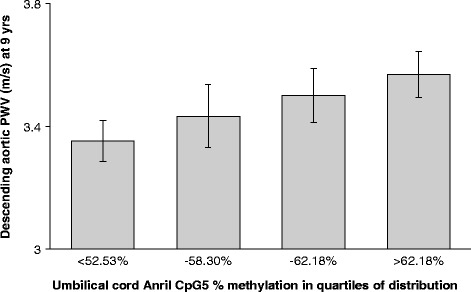
Pulse wave velocity (PWV) vs methylation of ANRIL promoter CpG5. DNA methylation levels were determined via pyrosequencing. The *x*-axis shows quartiles of methylation; values are means + SEM. *N* = 132

ANRIL promoter CpG5 methylation was positively associated with PWV (*p* = 0.037) (Fig. 2, Table 3); the association remained after controlling for child’s total fat at 8 years, sex, age and maternal smoking; adjusted *p* = 0.026 (total variance explained 3.2 %). Childhood total fat and %fat at 8 years and maternal smoking were not independent predictors of PWV.

**Table 3:** Pulse wave velocity (m/s) at age 9 years in relation to ANRIL DNA methylation

CpG	Hg19 coordinates	*β* value	*p* value	Adjusted *p*
1	chr9: 21993721	0.0089	0.803	0.770
2	chr9: 21993697	0.0173	0.622	0.592
3	chr9: 21993694	−0.053	0.357	0.723
4	chr9: 21993654	0.0016	0.967	0.621
5	chr9: 21993645	0.0663	0.037	0.026
6	chr9: 21993638	0.0207	0.583	0.345
7	chr9: 21993629	0.0154	0.736	0.659
8	chr9: 21993603	0.0258	0.531	0.322
9	chr9: 21993583	0.0828	0.176	0.087

*β* values represent a 10 % change in methylation. DNA methylation levels were determined via pyrosequencing. Linear regressions were used to compare methylation and pulse wave velocity. Adjusted *p* values are adjusted for age, sex, total fat mass at 8 years and mother smoking during pregnancy

### Blood pressure is not associated with ANRIL DNA methylation

ANRIL methylation showed no association with childhood systolic or diastolic blood pressure.

MatInspector was used to examine the DNA sequence surrounding CpGs1, 2 and 5 to determine if these CpG dinucleotides aligned with any known transcription factor (TF) binding sites. This identified ten different potential TFs with a core similarity score >0.8, including SMAD, PPAR, ERE, KLF, HIF and GATA (Additional file 4: Figure S1).

All the associations with perinatal ANRIL promoter methylation were independent of neighbouring genetic variants (data not shown).

## Discussion

Lower DNA methylation at two adjacent CpG dinucleotides within the promoter of ANRIL was associated with faster heart rate, independent of potential confounders. Elevated heart rate is an independent risk factor for CHD [20, 21], a predictor of cardiovascular mortality [22], and is associated with reduced longevity, even in those without pre-existing cardiovascular conditions [22]. Mean resting pulse rate has increased by up to 2 bpm among 9–11-year UK children over nearly 30 years [23]. This has been attributed to an increase in adiposity and decline in physical fitness among children; however, these findings suggest that the prenatal environment may also be an important determinant of later CHD risk and provides support for epigenetic processes in mediating the long-term consequences of the prenatal environment on CHD risk.

Higher DNA methylation at CpG5, which lies more distal to the transcriptional start site (TSS) of ANRIL, was associated with higher PWV—a marker for increased arterial stiffness that also indicates greater cardiovascular risk. This difference in the direction of associations observed between the methylation of the individual CpG sites within the ANRIL promoter and CHD risk factors may reflect the fact that gene promoter DNA methylation often influences expression through the modulation of transcription factor binding [24], and while some studies have shown that the methylation status of CpG sites can be closely aligned to that of their neighbours, especially within a CpG island, developmentally induced changes are often CpG site specific [25]. Consistent with this, an in silico analysis predicted that distinct transcription factors may bind across the different CpG loci associated with heart rate and PWV, respectively, suggesting that the CpG sites may be independently regulated. Across CpGs 1 and 2, potential TF binding sites include SMAD and KLF, which are involved in heart development and the proliferation of cardiomyocytes [26–28], as well as PPAR, linked to atherosclerosis [29] and ERE, which is important for angiogenesis and modulation of vascular smooth muscle cells (VSMC) [30]. CpG 5 is close to a potential TF binding site for HIF—an important regulator of oxygen homeostasis [31], as well as a binding site for GATA, which is critical for heart development [32]. While transcription factor binding will need to be validated experimentally, this does suggest distinct roles for CpG 1 and 2 compared to CpG5 and is compatible with the purported role of ANRIL transcripts in VSMC [6].

The strengths of this study are the relative large number of participants with detailed phenotypic cardiovascular characterisation. There are some limitations; this is only a preliminary study, and the results need to be replicated in other studies to confirm our findings. Secondly, we analysed methylation in umbilical cord samples, and DNA methylation patterns are often tissue-specific; however, the umbilical cord does contain a high proportion of fetal vascular tissue, and so is likely to be relevant to cardiovascular disease risk phenotypes studied here. A third limitation is that although all known SNPs within 45 bp of the CpGs sites studied were excluded by direct sequencing, without genome-wide sequencing, it is not possible to exclude the effect of distant SNPs. Fourth, we did not have longitudinal data, so cannot ascertain whether methylation of ANRIL is a driver of altered heart rate or PWV or a consequence of these changes. It would be interesting to follow the methylation of ANRIL with regard to CHD risk factors during childhood and later life to determine how ANRIL methylation tracks with these factors over time, and it would also be interesting to examine ANRIL expression levels. Nevertheless, the finding that altered methylation of CpGs within the promoter of ANRIL, a long non-coding RNA previously linked to CHD risk through GWAS, is associated with predictors of CHD risk in childhood suggest that differential methylation of this region maybe a marker which could be used to identify those individuals at increased risk of CHD in early life.

## Conclusion

In summary, our findings suggest that altered epigenetic regulation of ANRIL, a gene strongly linked to CHD by genome-wide association studies, is associated with alterations in both heart rate and arterial stiffness. Cardiovascular disease is often identified late in its pre-clinical phase, necessitating intervention to manage disease progression. Thus, the identification of perinatal epigenetic marks that are predictive of later disease risk represents an opportunity to identify those individuals who are at greater risk of subsequent disease in early life and a means to monitor the effectiveness of preventative interventions.

## Additional files

Additional file 1: Table S2. Primers used for methylation analysis of ANRIL promoter. (DOCX 13 kb) Additional file 2: Table S1. Observed DNA methylation ranges for CpG dinucleotides quantified by pyrosequencing. CpG coordinates are provided in Hg19. Distance from transcriptional start site (TSS) in base pairs. *N* = 132. (DOCX 14 kb) Additional file 3: Table S3. Correlation of the methylation levels between CpG sites. Pairwise Spearman correlation. (DOCX 15 kb) Additional file 4: Figure S1. Predicted consensus transcription factor binding sequences. MatInspector was used to examine the DNA sequence around CpGs 1, 2, and 5 to identify potential binding sites for transcription factors using MatInspectors core/vertebrate transcription factor database. Core sim. (core similarities) and Matrix sim. (matrix similarities) are scored out of 1. Results with Core Sim. scores >0.8 are shown. Core sim. Refers to base pair matching for the core consensus sequence (underlined + BOLD in transcription factor sequences) while Matrix sim. Refers to overall matching across the full binding site. (DOCX 17 kb)

## Abbreviations

CHD: Coronary heart disease
MRI: Magnetic resonance imaging
PWV: Pulse wave velocity
TF: Transcription factor
TSS: Transcriptional start site
VSMC: Vascular smooth muscle cells
