# Supplementary material for: DNA methylation at birth within the promoter of ANRIL predicts markers of cardiovascular risk at 9 years
Source: Clin Epigenetics. 2016 Sep 2;8(1):90. doi: 10.1186/s13148-016-0259-5 (PMC5010744; doi:10.1186/s13148-016-0259-5)
Supplement: Additional file 1: Table S2. — Primers used for methylation analysis of ANRIL promoter. (DOCX 13 kb) [file 13148_2016_259_MOESM1_ESM.docx]

**Supplementary Table 2. Primers used for methylation analysis of ANRIL promoter**

| **Primer** | **Sequence** |
| --- | --- |
| CDKN2A 1-3 F | AGTAGGAAAGGTGTATTTTAAGTATATTT |
| CDKN2A 1-3 S | AGAATTATTGTTAATTATTTAAGTT |
| CDKN2A 4-9 F | TGGGGAGAATTATTGTTAATTATTTAAGTT |
| CDKN2A 4-9 S | TAGGAGAGTGGAGGA |
| CDKN2A 8-9 S | GTAGGTAGAGATTTTTTGAAATGT |
| CDKN2A 1-3 R | TATCTCACCAATCCTCCACTCTCCTAAA |
| CDKN2A 4-9 R | AAAAACCCATTTCCCTATTAACTACA |
