# Supplementary material for: DNA methylation at birth within the promoter of ANRIL predicts markers of cardiovascular risk at 9 years
Source: Clin Epigenetics. 2016 Sep 2;8(1):90. doi: 10.1186/s13148-016-0259-5 (PMC5010744; doi:10.1186/s13148-016-0259-5)
Supplement: Additional file 2: Table S1. — Observed DNA methylation ranges for CpG dinucleotides quantified by pyrosequencing. CpG coordinates are provided in Hg19. Distance from transcriptional start site (TSS) in base pairs. N = 132. (DOCX 14 kb) [file 13148_2016_259_MOESM2_ESM.docx]

**Supplementary Table 1. Observed DNA methylation ranges for CpG dinucleotides quantified by pyrosequencing.** CpG coordinates are provided in Hg19. Distance from transcriptional start site (TSS) in base pairs. **N = 132**

| **CpG** | **coordinates** | **Distance from ANRIL TSS** | **N** | **median (5th, 95th percentile)** |
| --- | --- | --- | --- | --- |
| 1 | chr9: 21993721 | -1069 | 127 | 71.9 (48.1, 81.5) |
| 2 | chr9: 21993697 | -1093 | 124 | 67.7 (48.6, 78.6) |
| 3 | chr9: 21993694 | -1096 | 110 | 55.4 (39.5, 62.0) |
| 4 | chr9: 21993654 | -1136 | 136 | 70.7 (53.2, 80.4) |
| 5 | chr9: 21993645 | -1145 | 136 | 58.7 (39.4, 69.6) |
| 6 | chr9: 21993638 | -1152 | 136 | 71.3 (52.9, 80.0) |
| 7 | chr9: 21993629 | -1161 | 135 | 58.4 (42.8, 68.0) |
| 8 | chr9: 21993603 | -1187 | 122 | 73.3 (59.4, 81.3) |
| 9 | chr9: 21993583 | -1207 | 118 | 60.5 (50.4, 67.5) |
