# Supplementary material for: DNA methylation at birth within the promoter of ANRIL predicts markers of cardiovascular risk at 9 years
Source: Clin Epigenetics. 2016 Sep 2;8(1):90. doi: 10.1186/s13148-016-0259-5 (PMC5010744; doi:10.1186/s13148-016-0259-5)
Supplement: Additional file 3: Table S3. — Correlation of the methylation levels between CpG sites. Pairwise Spearman correlation. (DOCX 15 kb) [file 13148_2016_259_MOESM3_ESM.docx]

**Supplementary Table 3. Correlation of the methylation levels between CpG sites.** Pairwise Spearman correlation.

|  | **CpG1** | **CpG2** | **CpG3** | **CpG4** | **CpG5** | **CpG6** | **CpG7** | **CpG8** | **CpG9** |
| --- | --- | --- | --- | --- | --- | --- | --- | --- | --- |
| **CpG1** | **1** |  |  |  |  |  |  |  |  |
| **CpG2** | 0.820 | **1** |  |  |  |  |  |  |  |
| **CpG3** | 0.820 | 0.761 | **1** |  |  |  |  |  |  |
| **CpG4** | 0.514 | 0.601 | 0.500 | **1** |  |  |  |  |  |
| **CpG5** | 0.585 | 0.668 | 0.481 | 0.778 | **1** |  |  |  |  |
| **CpG6** | 0.616 | 0.648 | 0.576 | 0.801 | 0.828 | **1** |  |  |  |
| **CpG7** | 0.578 | 0.638 | 0.497 | 0.793 | 0.805 | 0.791 | **1** |  |  |
| **CpG8** | 0.518 | 0.631 | 0.422 | 0.634 | 0.668 | 0.741 | 0.738 | **1** |  |
| **CpG9** | 0.479 | 0.587 | 0.470 | 0.587 | 0.627 | 0.629 | 0.660 | 0.705 | **1** |
