# Supplementary material for: DNA methylation at birth within the promoter of ANRIL predicts markers of cardiovascular risk at 9 years
Source: Clin Epigenetics. 2016 Sep 2;8(1):90. doi: 10.1186/s13148-016-0259-5 (PMC5010744; doi:10.1186/s13148-016-0259-5)
Supplement: Additional file 4: Figure S1. — Predicted consensus transcription factor binding sequences. MatInspector was used to examine the DNA sequence around CpGs 1, 2, and 5 to identify potential binding sites for transcription factors using MatInspectors core/vertebrate transcription factor database. Core sim. (core similarities) and Matrix sim. (matrix similarities) are scored out of 1. Results with Core Sim. scores >0.8 are shown. Core sim. Refers to base pair matching for the core consensus sequence (underlined + BOLD in transcription factor sequences) while Matrix sim. Refers to overall matching across the full binding site. (DOCX 17 kb) [file 13148_2016_259_MOESM4_ESM.docx]

**Supplementary Figure 1. Predicted consensus transcription factor binding sequences.** MatInspector was used to examine the DNA sequence around CpGs 1, 2, and 5 to identify potential binding sites for transcription factors using MatInspectors core/vertebrate transcription factor database. Core sim. (core similarities) and Matrix sim. (matrix similarities) are scored out of 1. Results with Core Sim. scores >0.8 are shown. Core sim. Refers to base pair matching for the core consensus sequence (underlined+BOLD in Transcription Factor sequences) while Matrix sim. Refers to overall matching across the full binding site.

CpG1

GGAGGCTGGGGAGAAAAAAGGC***CG***CCTCCAGAAAACTTAGATGGTTAGCAATAA

gccgc**CTCC**ag SMAD

| **Matrix Family** | **Detailed Family Information** | **Core sim.** | **Matrix sim.** |
| --- | --- | --- | --- |
| SMAD | Vertebrate SMAD family of transcription factors | 1.00 | 0.94 |

CpG2

CTGGAAGGTGGGAGAGGGTGACCCCGC***CG***GGAGGCTGGGGAGAAAAA

agg**TGGG**agagggtgaccccgcc PPAR

ggtgggagagggt**GACC**ccgccg ERE

gtg**ACCC**cgccgggagg KLF

gggtgaccccgcc**GGGA**ggctgg NOLF

cgccgggaggct**GGGG**agaaaaa ZF02

| **Matrix Family** | **Detailed Family Information** | **Core sim.** | **Matrix sim.** |
| --- | --- | --- | --- |
| PPAR | Peroxisome proliferator activated receptor homodimers | 0.86 | 0.71 |
| ERE | Estrogen response elements | 1.00 | 0.93 |
| KLF | Krueppel like transcription factor | 1.00 | 0.89 |
| NOLF | Neuron-specific olfactory factor | 1.00 | 0.88 |
| ZF02 | C2H2 zinc finger transcription factors 2 | 1.00 | 0.99 |

CpG5

GTCGCTGCCTGCGTGCCC***CG***TATCTCACGGGTCCTCCACTCTC

gctgcctg**CGTG**ccccg HIFF

gtcgctgcct**GCGT**gccccgtatct AHRR

gcccc**GTAT**ctca DMTF

ccccg**TATC**tcac GATA

| **Matrix Family** | **Detailed Family Information** | **Core sim.** | **Matrix sim.** |
| --- | --- | --- | --- |
| HIFF | Hypoxia inducible factor, bHLH/PAS protein family | 1.00 | 0.93 |
| AHRR | AHR-arnt heterodimers and AHR-related factors | 1.00 | 0.96 |
| DMTF | Cyclin D binding myb-like transcription factor | 0.87 | 0.85 |
| GATA | GATA binding factors | 1.00 | 0.99 |
